# A method to enhance the use of interaural time differences for cochlear implants in reverberant environments

**DOI:** 10.1121/1.4960572

**Published:** 2016-08

**Authors:** Jessica J. M. Monaghan, Bernhard U. Seeber

**Affiliations:** Medical Research Council Institute of Hearing Research, Nottingham, United Kingdom

## Abstract

The ability of normal-hearing (NH) listeners to exploit interaural time difference (ITD) cues conveyed in the modulated envelopes of high-frequency sounds is poor compared to ITD cues transmitted in the temporal fine structure at low frequencies. Sensitivity to envelope ITDs is further degraded when envelopes become less steep, when modulation depth is reduced, and when envelopes become less similar between the ears, common factors when listening in reverberant environments. The vulnerability of envelope ITDs is particularly problematic for cochlear implant (CI) users, as they rely on information conveyed by slowly varying amplitude envelopes. Here, an approach to improve access to envelope ITDs for CIs is described in which, rather than attempting to reduce reverberation, the perceptual saliency of cues relating to the source is increased by selectively sharpening peaks in the amplitude envelope judged to contain reliable ITDs. Performance of the algorithm with room reverberation was assessed through simulating listening with bilateral CIs in headphone experiments with NH listeners. Relative to simulated standard CI processing, stimuli processed with the algorithm generated lower ITD discrimination thresholds and increased extents of laterality. Depending on parameterization, intelligibility was unchanged or somewhat reduced. The algorithm has the potential to improve spatial listening with CIs.

## I Introduction

Cochlear implants (CIs) restore hearing function in the severely and profoundly deaf, providing many users with high levels of speech understanding, particularly when background noise is low. For most listeners, however, performance is substantially degraded in even moderate levels of background noise, and CI users with only one implant are unable to access spatial information that might help them resolve complex acoustic scenes where strategies for “cocktail party” listening are required.

**Fig. 1:**
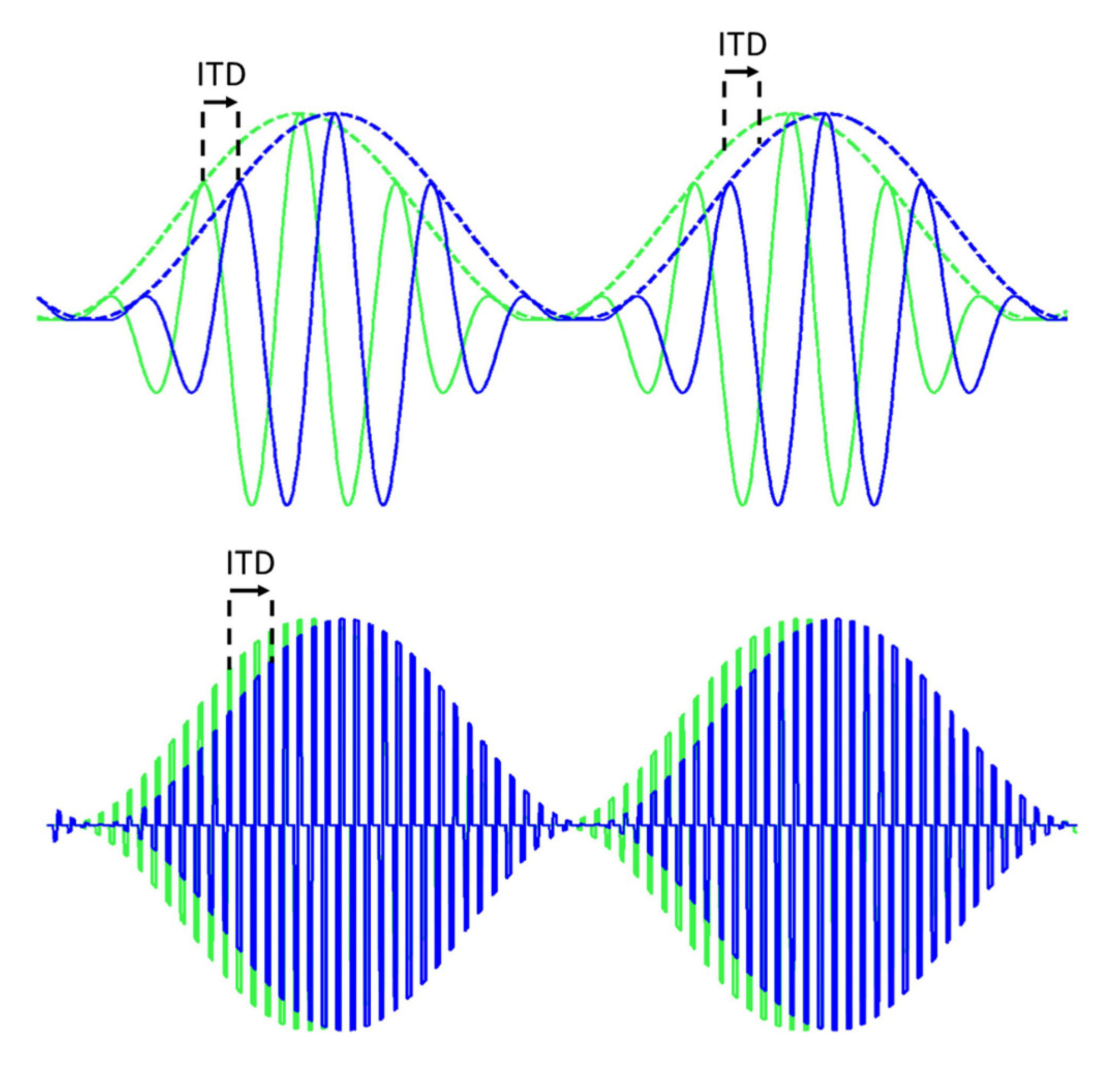
(Color online) ITDs in acoustic signals (top) and electrical signals in CIs (bottom). In most current CI processing the fine-structure present in the acoustic signal is replaced by a fixed rate electrical pulse train so that the ITD is conveyed only in the envelope.

To this end, bilateral cochlear implantation offers the potential for improving listening performance in noisy conditions, including through the restoration of binaural localization cues. Nevertheless, several technical (and likely physiological) limitations must be overcome before the promise of true spatial hearing in bilateral CI can be fully realized. Binaural cues for localization comprise differences in the timing (interaural time differences; ITDs) and level (interaural level differences; ILDs) of the sound at each ear. In acoustic hearing, ITDs are conveyed through the temporal fine structure (TFS), as well as through the time-varying amplitude, or temporal envelope, of modulated sounds. In electrical hearing, however, the very high stimulation rates employed by most current CI devices convey temporal information (including ITD information) only in the modulated envelope of sounds; electrical pulses used to stimulate the electrode array are presented at a fixed, high rate completely unrelated to the sound’s TFS (see Fig. 1). At these high rates, ITD information carried in the timing of individual stimulation pulses is inaccessible to CI users (Laback *et al*., 2007). This restriction, of itself, does not prevent the use of ITD information for localization in electric hearing; as for normal-hearing (NH) listeners, CI users are sensitive to envelope ITD cues. Using direct stimulation with modulated electrical pulse trains, van Hoesel and Tyler (2003) found ITD discrimination thresholds in bilateral CI users to approach those of NH listeners for envelope ITDs (~100 *μ*s; Bernstein and Trahiotis, 2002; Monaghan *et al.*, 2015), suggesting a similar accuracy of the neural representation of temporal envelopes in electric hearing. Nonetheless, localization experiments conducted under free-field conditions demonstrate that while some bilateral CI users show relatively high performance in localization tasks, they predominantly exploit ILDs in their judgments, rather than ITDs, even under anechoic listening conditions where ITD information is available in the absence of potentially confounding echoes or interfering sources (van Hoesel and Tyler, 2003; Seeber and Fastl, 2008). In a simulated reverberant environment, Kerber and Seeber (2013) showed that the root-mean-square (RMS) localization error increased for all CI users relative to localization in an anechoic environment. In five of seven CI users, the increase was larger than 10°, and in two CI users it exceeded 20°. The environment simulated was a moderately reverberant room, indicating that, for the majority of CI listeners, reverberation will adversely affect localization of sounds in everyday listening environments. However, it appears that particularly in more complex, reverberant listening conditions, CI users can benefit from additional information available from envelope ITDs to improve their localization performance (Kerber and Seeber, 2013).

In studies of NH listeners, envelope ITDs are found to be most accessible for stimuli with rapid attacks, such as pulse trains, for which ITD discrimination thresholds can be lower than 100 *μ*s (Laback *et al*., 2011). However, for the more slowly varying amplitude envelopes of speech sounds—the most relevant stimuli for real-world listening—threshold ITDs are likely to be higher, a consequence of the shallow rising flanks of the stimulus envelope conveying ITD information. For NH listeners, envelope ITD thresholds are lowest when onset gradients are >6 dB/ms (Klein-Hennig *et al.*, 2011; Laback *et al.*, 2011; Monaghan *et al.*, 2013). This increase in ITD thresholds will be exacerbated in realistic environments where reflections from walls and other hard surfaces tend to have a low-pass filtering effect on the signal (rendering stimulus envelopes even shallower), and reduce the binaural coherence (the degree of similarity of the signal between the ears). Either of these factors alone (Klein-Hennig *et al.*, 2011; Monaghan *et al.*, 2013) is sufficient to degrade sensitivity to ITDs conveyed in temporal envelopes and, consistent with this, discrimination of envelope ITDs is highly susceptible to room reverberation (Monaghan *et al.*, 2013). Nevertheless, these, and other studies (e.g., Bernstein and Trahiotis, 2009), indicate that high performance (i.e., low ITD thresholds) is possible for NH listeners if the flanks of the temporal envelope are sufficiently steep, envelope modulation is sufficiently deep, and the interaural coherence is high. Since ITD processing in CI users appears to suffer from the same limitations as envelope ITDs processing in NH listeners (Laback *et al.*, 2011), it may be expected that signal-processing strategies that enhance these envelope characteristics would help CI users to extract ITDs conveyed in the envelopes of real-world sounds more effectively. Here, we employed simulations of listening with bilateral CIs in rooms in order to investigate whether envelope enhancement algorithms can reduce the detrimental effects of reverberation on spatial hearing abilities and speech understanding.

In automatic speech recognition (ASR) and computational auditory scene analysis (CASA), the problem of reverberation is tackled by de-reverberation techniques, which attempt to reconstruct the direct sound and remove reflections. One method used for de-reverberation in ASR and CASA is blind source separation, in which two or more sources are inferred by the signal arriving at two or more microphones. This technique was employed by Kokkinakis and Loizou (2008) to produce a bilateral CI strategy that improved users’ speech recognition performance in noise and reverberation relative to the standard processing. Here, we present a novel algorithm that relies on selective enhancement of ITD information through the sharpening of stimulus envelopes. The novelty of this approach is that rather than trying to remove the extraneous or misleading information in the sound signals, the clean (ITD) information is instead made more perceptually salient. The goal, therefore, is to optimize the input such that perceptual cues are more salient rather than to solve the dereverberation problem (as would be required for CASA), potentially producing fewer artifacts. The new approach does not attempt to reduce the reverberation and, hence, can be combined with classic, subtractive de-reverberation techniques. Similar approaches were previously followed to enhance pitch perception in monaural CI processing. For example, Green *et al.* (2004) enhanced fundamental frequency (F0) information in the stimulus envelope, demonstrating a benefit for prosody identification, by modulating the envelopes by a sawtooth waveform with a frequency of F0. The “F0mod” strategy (Geurts and Wouters, 2001) increases the modulation depth of envelope fluctuations at F0 by subtracting a low-pass filtered version of the envelope from the original envelope. This strategy improved lexical tone perception in Mandarin Chinese (Milczynski *et al.*, 2012). The benefit was increased when synchronizing temporal modulation across frequency bands and extended to pitch ranking and melody recognition (Milczynski *et al.*, 2009). These results are promising, despite the limited salience of temporal pitch cues (Moore, 2003). Because the processes of pitch and ITD discrimination both require a sharp neural representation of the temporal information in the monaural signals, and since this representation is enhanced by steeper envelope flanks, processing strategies that sharpen the envelope of the direct sound would likely enhance both ITD discrimination and pitch perception. Evidence from CI listeners with contralateral acoustic hearing suggests that localization in anechoic space can be improved with such an approach (Francart *et al*., 2014).

Pitch enhancement techniques have been directed toward enhancing a single F0 in quiet. The task of enhancing envelope ITD information in reverberation, however, is made more difficult by the fact that the envelopes of sounds arriving at the ears convey not only the ITD of the source (direct sound), but also the ITDs of reflections. Here, the aim of envelope ITD enhancement is to increase saliency of information about the source without increasing the saliency of reflections. Indiscriminate sharpening of envelopes is thus unlikely to improve ITD discrimination in reverberation, since early reflections could otherwise stand out or diffuse late reverberation could blur the auditory object. Therefore, envelope sharpening should be applied selectively, such that it enhances only those parts of the envelope that convey the ITD generated by the source. Several approaches can be used to determine those moments when the direct signal ITD dominates, amongst them methods based on estimation of interaural correlation (e.g., Faller and Merimaa, 2004), auditory scene analysis (e.g., Wittkop *et al.*, 1997), or blind source separation (e.g., Kokkinakis and Loizou, 2008). The method described here used prior knowledge of either the ITD corresponding to the source direction or the direct-to-reverberant ratio (DRR) of the signal to identify these moments. The sharpening was then achieved by steepening the selected envelope peaks by increasing both the gradient of the rising flanks and the modulation depth of the envelope.

## II General Methods

### A Overview of the onset enhancement approach

**Fig. 2:**
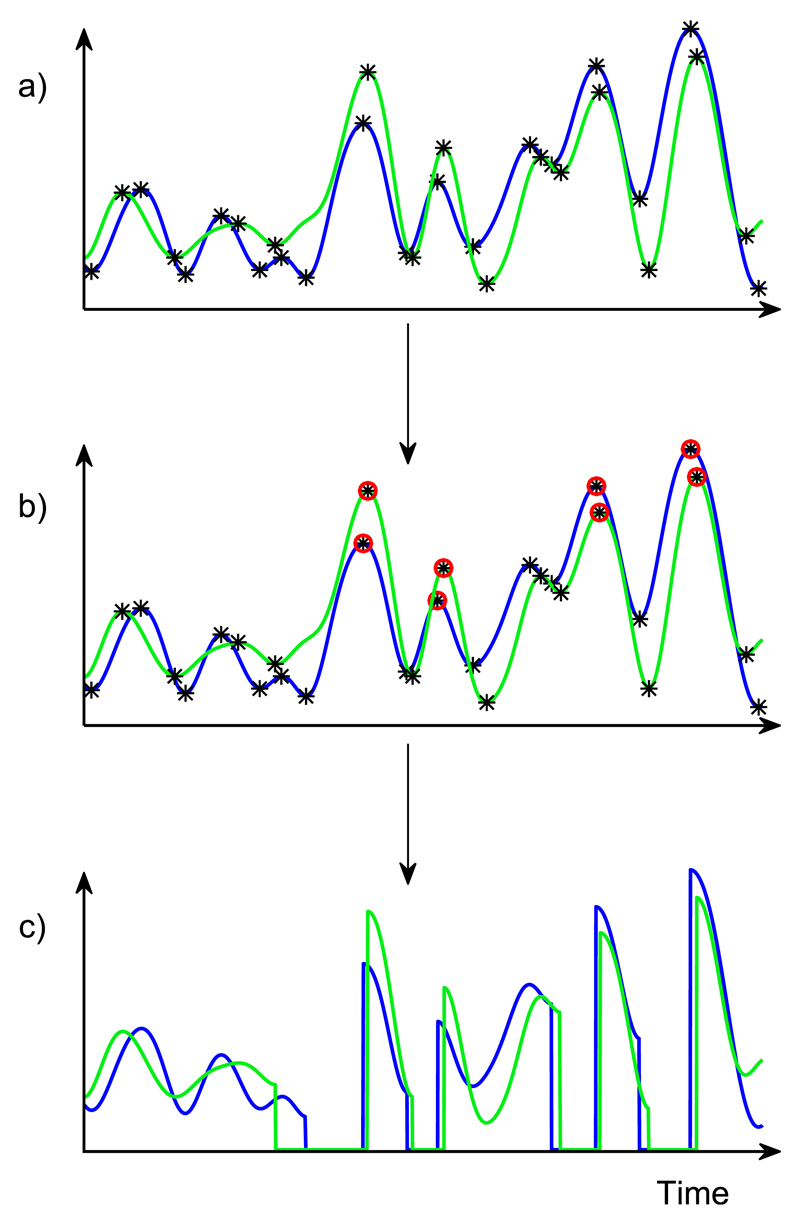
(Color online) Schematic of the envelope sharpening procedure. (a) After extracting the envelope in frequency bands, the temporal position of all the peaks and troughs of the envelope are identified. (b) The envelope properties around each peak are assessed and the peak is selected for sharpening only if they meet the ITD or DRR-criterion. (c) Selected peaks are sharpened by setting the envelope amplitude to zero from the selected peak to the preceding trough.

**Fig. 3:**
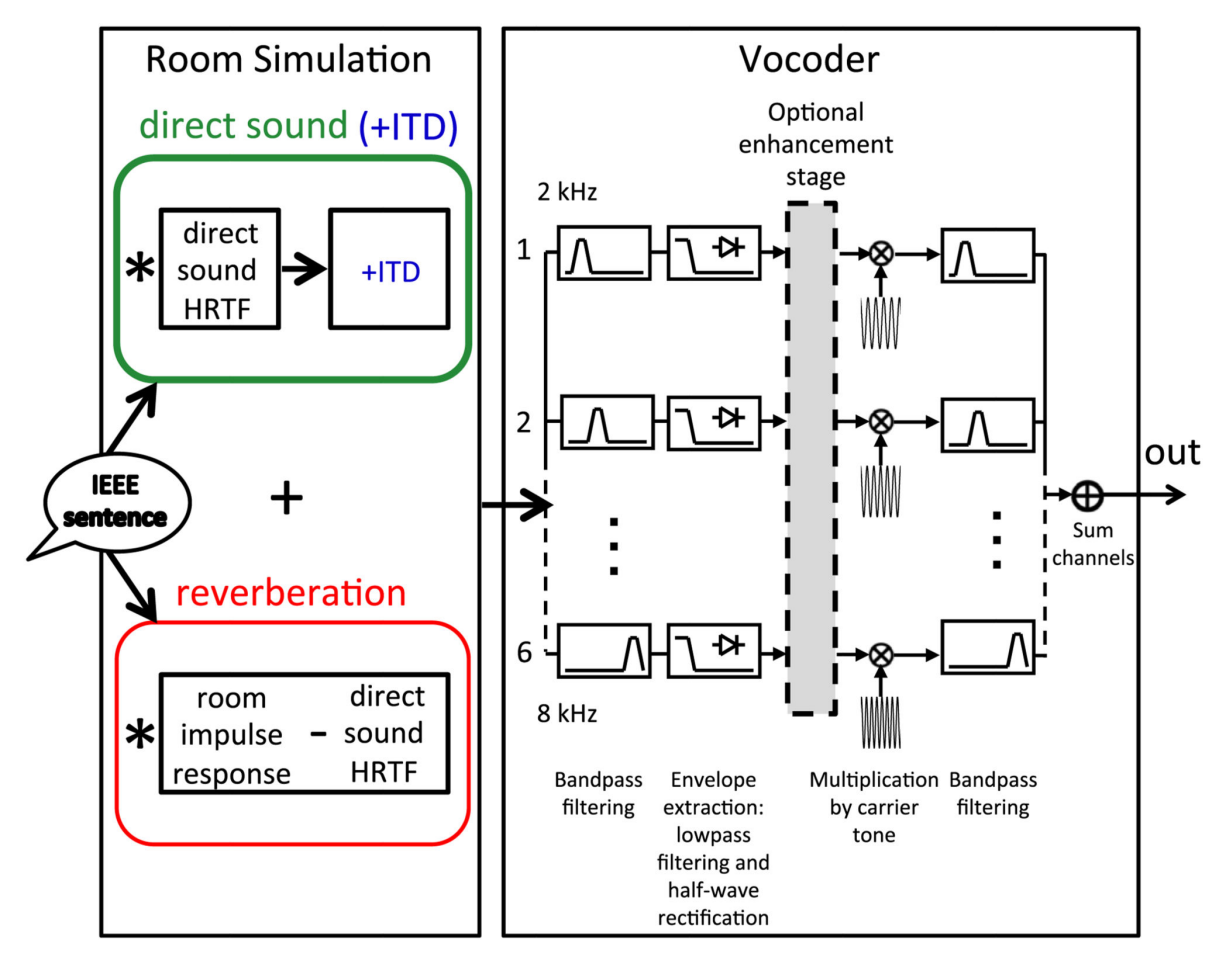
(Color online) Stimulus processing for experiments 1–3. Sentences from the IEEE sentence corpus were convolved with the reverberant and direct sound parts of the BRIR. ITDs to be tested were applied to the direct sound part only before addition of the reverberant sound. The simulated reverberant sentence is then vocoded with a six-band-vocoder in experiments 1 and 2 or an eight-band-vocoder in experiment 3, both restricted to high frequencies (lowest corner frequency 1.265 kHz). After bandpass filtering, envelopes are extracted in each band by half-wave rectification and low-pass filtering. Then, envelope enhancement is performed depending on the condition, and the enhanced envelopes are used to modulate sinusoidal carriers of the center frequency of the band. The bands are bandpass-filtered and summed to create the vocoded stimulus.

In CI processing, the incoming audio signal is bandpass filtered into a number of frequency bands and the envelope is extracted in each band. The onset enhancement takes place on extracted envelopes (see Sec. II B 2, Figs. 2 and 3). The peaks and troughs are identified from the maxima and minima of the envelope by calculating the zero-crossings of the first derivative of the envelope. Peaks are enhanced selectively when they meet certain criteria related to the relative strength of the direct sound.

Two methods are proposed for identifying parts of the waveform dominated by the direct sound. The first method uses the instantaneous ITD of the envelope and is referred to hereafter as ITD-based enhancement. It assumes that when the instantaneous ITD of the signal matches the ITD of the direct sound, the signal is dominated by the direct sound. The second method uses the instantaneous DRR of the envelopes and is referred to hereafter as DRR-based enhancement. The DRR is the difference between the direct sound and all reverberant sound energy in dB, i.e., the log of the ratio of the sound energy arriving directly to the listener from the source and the sound arriving after being reflected from the walls. When the instantaneous DRR of the signal is positive, the signal is, necessarily, dominated by the direct sound.

Both of these methods require some knowledge about the sound source and the reverberation, either of the ITD corresponding to the sound-source location or of the DRR of the sound arriving at the listener. In practice these measures would have to be estimated from the sound signal, but prior knowledge is used here to provide an estimate of the ideal performance of the method without confounding this estimate with the errors associated with the DRR or ITD estimation process itself.

For the DRR-based method, two conditions have to be met for an envelope peak to be enhanced. The first condition ensures that peaks are enhanced if the direct sound dominates the reverberation (by definition, DRR > 0). These peaks are assumed to more reliably carry the ITD of the source. To this end, the DRR was calculated on a sample-by-sample basis for each ear. A peak was selected if the DRR was positive for at least one sample in a region of ±3 samples (with three samples at 44.1 kHz corresponding to 68 *μ*s) from that peak (at the same ear). Second, there must be a peak in the signal at the other ear that meets the first criterion within ±2.27 ms of the peak. This second criterion was implemented to reduce the probability of choosing a peak for enhancement that had no matching peak on the other ear, thereby transmitting a spurious value of the source ITD. For the ITD-based method, the ITD between the two peaks must be equal to the source ITD ±0.3 ms if a pair of envelope peaks (one at each ear) is to be enhanced. The ±0.3-ms tolerance was introduced to increase the number of peaks selected. This provides for a “beam” of enhancement, symmetric around the source location. If the tolerance is too high, the enhancement will not be specific enough, but if the tolerance is too low, too few peaks will be selected, and the enhancement will have little effect. The value of ±0.3 ms was chosen since the number of peaks selected was of the same order as those for the DRR-based method (see Fig. 5). For both methods, if the chosen condition was met for a particular envelope peak, the rising flank was sharpened by setting the amplitude of the envelope to zero between the selected peak and the preceding trough [Fig. 2(c)]. This method of sharpening is based on the study of Monaghan *et al.* (2013), which indicates no apparent limit to the improvement in threshold with increasing steepness, at least over the range tested (2.4 to 30 dB/ms). Setting the rising flank to zero (rather than, for example, its original minimum) also increases the modulation depth of these segments of the envelope. As demonstrated by Monaghan *et al.* (2013), the benefit of steep onsets cannot be realized if the modulation depth is too low. The selection and sharpening of envelope peaks is illustrated in Fig. 2.

### B Evaluation of the onset enhancement approach using a vocoder

The methods for enhancement of envelope peaks were assessed in NH listeners, employing a special vocoding approach to simulate aspects of listening through bilateral CIs. Evaluation was conducted in virtual acoustic space simulating listening in a room.

The stimuli were designed to provide an approximation to listening to speech in a room using bilateral CIs. To this end, speech stimuli from the IEEE test sentence corpus (Rothauser *et al.*, 1969) were convolved with a binaural room impulse response (BRIR) and then vocoded. The vocoder limited information to high-frequency bands (like high-pass filtering) so that only envelope ITDs could be used by the NH listeners to prevent interference from fine-structure ITDs (Seeber and Hafter, 2011). Figure 3 shows a schematic of the stimulus processing.

#### 1 Spatial processing

The reflections arising from a source in an empty rectangular room were calculated using custom-made software based on the image-source method (Seeber *et al.*, 2010). The image-source method models specular reflections of the sound source as additional virtual sources. The simulation is calculated up to a maximum reflection order (20) whereby only valid and visible image sources are auralized.

The simulated room was designed to be 4.7 m × 6.8 m in plan and 2.5 m high and the sound source was located 1 m from the shorter wall, 0.7 m from the long wall, and at a height of 1.4 m. The source was modeled as being directed toward the listener using the mouth directivity functions measured by Flanagan (1960). The dimensions of the room and the position of the source were chosen to be favorable in terms of avoiding room modes. The absorption coefficients of the walls, floor, and ceiling were chosen to simulate an unfurnished, carpeted room with gypsum-cardboard walls. The reverberation radius of the room, the distance at which the energy from the direct sound was equal to the energy of all of the reflections arriving at the listener, was calculated to be 0.81 m. The *T*_30_ of the room, the time required for reflections of a direct sound to decay by 60 dB relative to the level of the direct sound, calculated as double the time for a decay from −5 to −35 dB, was 400 ms. Using HRTFs (head-related transfer functions) from participant “Bru” taken from the AUDIS project (Blauert *et al.*, 1998), five BRIRs were calculated for a receiver separated from the source by distances of 0.5 m, 1.0 m, 1.5 m, 2.0 m, and 2.5 m (for which distances the respective DRRs were 1.8, −4.0, −7.4, −9.7, and −11.4 dB) along a line orientated at 68° from the short wall. The receiver was modeled as always facing the source, i.e., the direct sound contained ITDs and ILDs near zero. The receiver was set at the same elevation as the source to avoid changes in elevation-related pinna cues that arise with increasing distance from the source. Each reflection was added as coming from the direction of the image source, including distance attenuation and delay for free path propagation plus frequency-dependent attenuation caused by the reflection on the walls.

The BRIRs were separated into the direct sound part (HRTF, attenuation, and delay) and a part containing all reflections. The direct sound—the sound arriving at the receiver directly from the source with no reflection—was calculated by convolving the stimulus with the HRTF for frontal incidence. Likewise, the reverberant sound—sound that had been reflected before arriving at the listener—was calculated by convolving the stimulus with the reverberation part of the impulse response. The ITDs to be discriminated were applied only to the direct sound and the reverberant sound was added with no additional ITD. This allowed the sensitivity to the ITD of the direct sound alone to be tested, while keeping the reverberant sound constant so that measurements would not be confounded by listeners detecting changes in reverberation. Note that the ILD of the direct sound was always zero, only the ITD was varied.

#### 2 Vocoding

To simulate CI processing the stimuli were filtered into bands and the envelopes extracted. For the two sets of enhanced conditions, the envelope enhancement algorithms were performed on the envelopes extracted from each frequency band, with the left and right channel in each frequency band being enhanced together. In these conditions, the enhanced envelopes were used to modulate sine-wave carriers, the frequencies of which were given by the center frequencies of the bands. Alternately, in the baseline condition, the sine carriers were modulated by the unprocessed envelopes. The bands were once again bandpass filtered (with the same filter characteristics as in the analysis stage) to restrict spectral splatter arising from the sharp envelope transitions introduced by the enhancement. Since this process limits the steepness of the final envelope, it was decided that this would represent the only limitation imposed on steepness. Such filtering would not be necessary in real CIs. The band outputs were summed and gated with simultaneous 50-ms Gaussian onset and offsets to reduce the effect of spurious ITDs in the onset and offset.

In order to determine how much benefit could be obtained by optimizing envelope shape alone, the acoustic simulations of CIs used here assumed an ideal configuration in which the electrodes are frequency matched across the ears. The simulation utilizes envelopes modulated on diotic carriers with frequencies above the limit for fine-structure ITD sensitivity, preventing an effect of carrier ITD (Seeber and Hafter, 2011). Translated to CIs, this mimics a high-rate carrier pulse train with no particular synchronization of stimulation pulses assumed for the algorithm to work.

For experiments 1 and 2 the vocoder used six contiguous frequency bands, at each ear between 2 kHz and 8 kHz with equal bandwidth in log Hz and center frequencies of 2.266, 2.854, 3.594, 4.525, 5.699, and 7.176 kHz. For experiment 3, to improve speech understanding, two extra bands were added with the same log bandwidth and center frequencies of 1.429 and 1.799 kHz. The vocoder processing first involved bandpass filtering the stimuli into the six bands with sixth-order Chebyshev filters. Envelopes were extracted from each band by half-wave rectification and low-pass filtering at 400 Hz with a 14th-order Chebyshev filter. Both the bandpass and low-pass filters were implemented as zero-phase filters by filtering the signal first forward and then backward in time with half of the stated filter. The cut-off frequency was set so that the envelope contains the strong modulations at the F0 (~200 Hz for female speech), which can be assumed to arise from the source. The steep cut-off removes spectral splatter generated by the process of half-wave rectification.

Stimuli were generated digitally with a sampling rate of 44.1 kHz using MATLAB, converted to analog with a 24-bit resolution converter built into a custom headphone amplifier and presented via headphones (Sennheiser HD 600, Wedemark, Germany) to the participant, who was seated in a double-walled, sound-attenuating room.

## III Experiment 1: Effect of Enhancement on ITD Discrimination

Experiment 1 was designed to test whether the algorithms described above could improve ITD discrimination thresholds in reverberation. This was motivated by the results of Monaghan *et al.* (2013) demonstrating lower ITD discrimination thresholds as the gradient of the envelope flanks was increased (provided the modulation depth is not too low).

### A Methods

Twenty IEEE sentences were processed as described in Sec. II B for the five virtual source-receiver distances. For each trial, one sentence was chosen pseudorandomly. An 880-ms segment of the vocoded sentence was extracted, 1 s from the end of the sentence to allow for the reverberation to have built up. The sentence segment was used as the stimulus and presented at 68 dB sound pressure level (SPL) (as measured after any enhancement was applied). An interaurally uncorrelated, equally exciting noise, low-pass filtered below 1.6 kHz (2 equivalent rectangular bandwidths below the 2 kHz corner frequency of the stimuli), was presented continuously at 64 dB SPL during stimulus presentation in order to mask potentially audible, low-frequency distortion products containing ITD information.

For all conditions, ITD discrimination thresholds were obtained using an adaptive three-down one-up procedure, returning the ITD that yields 79.4% correct responses (Levitt, 1971). The tracking algorithm employed used logarithmic step sizes, the initial ITD was 2000 *μ*s and the initial step size was a factor of 1.584 in ITD, decreasing to a factor of 1.122 after two reversals. Threshold estimates were determined by averaging over 12 reversals once this minimum step size was reached. Three threshold estimates were obtained for each condition from each listener. A two-interval, two-alternative forced-choice task was used to determine threshold. In the first interval, the stimulus was presented with half the ITD to be tested leading either to the left or the right with equal probability; in the second interval, the stimulus was presented with half the ITD leading to the opposite direction. Participants reported if the position of the intracranial auditory image moved left-right or right-left between intervals. This paradigm was used to counteract the effect of potential asymmetries in the participants’ perception of the stimulus position caused, for example, by lateral reflections. Feedback was given after each trial.

Five participants (four female) were assessed for all conditions. One participant was author J.J.M.M. In all experiments, the participants had no history of hearing disorders and had absolute thresholds within 20 dB hearing level (HL) at audiometric frequencies up to 8 kHz. Participants (except for J.J.M.M.) were paid for their participation.

### B Results and discussion

**Fig. 4:**
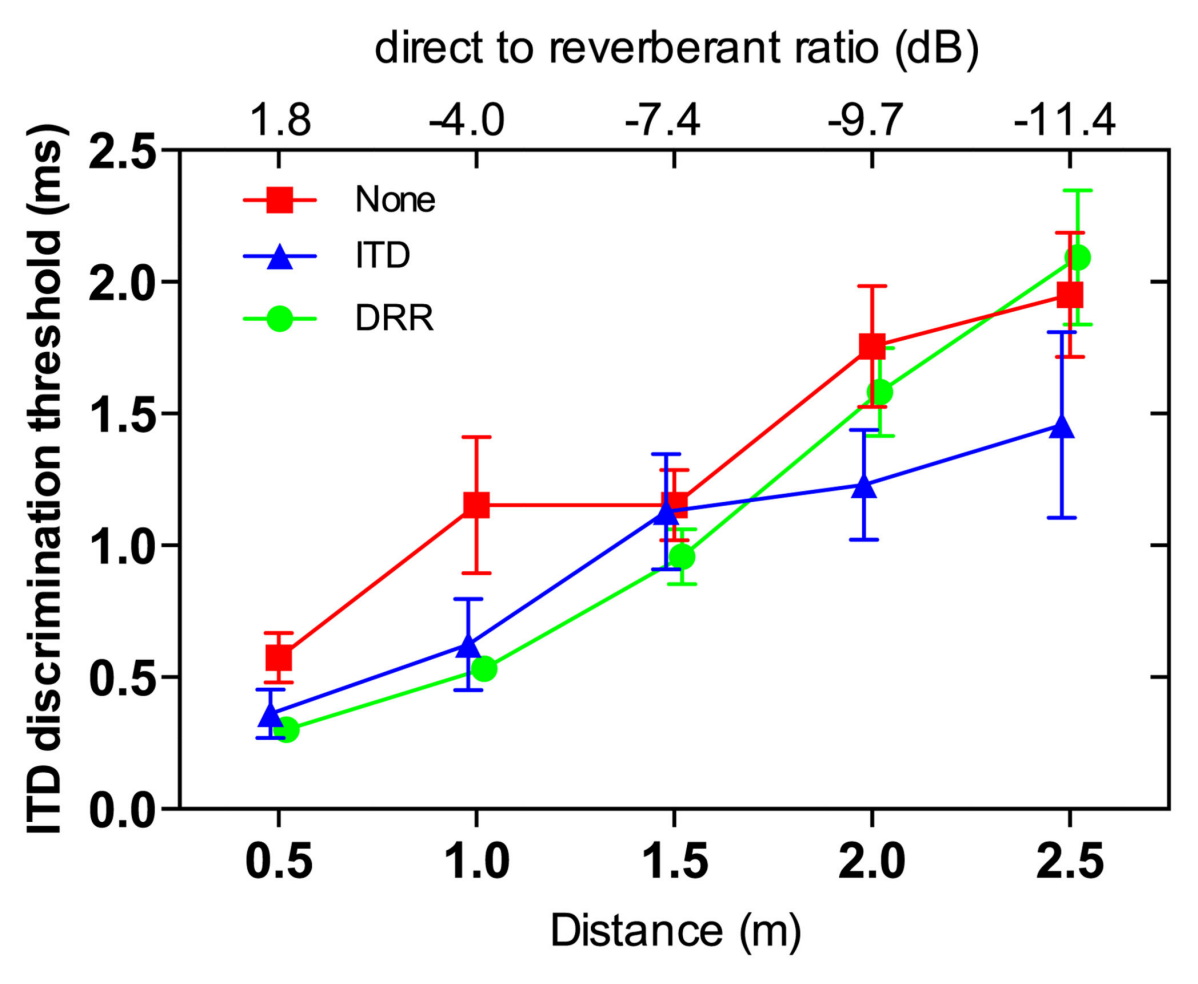
(Color online) Mean ITD discrimination thresholds across participants as a function of distance for DRR-based enhancement (circles and green line), ITD-based enhancement (triangles and blue line), and no enhancement (squares and red line). Error bars show standard errors.

Figure 4 shows the mean ITD discrimination thresholds across participants for each of the enhancement conditions (DRR-based enhancement-green line, ITD-based enhancement-blue line, and no enhancement-red line) as a function of distance. As distance from the source increased, ITD discrimination thresholds increased in all algorithm conditions. However, below 1.5 m, thresholds were lower with both enhanced conditions compared to the un-enhanced conditions. Close to the source, thresholds in both the ITD- and DRR-based conditions were approximately half those for the un-enhanced conditions, demonstrating a benefit from the enhancement algorithm for ITD sensitivity. Performance in the DRR- and ITD-based enhancement conditions were similar until the distance was >1.5 m, after which thresholds in the DRR-based enhancement conditions increased in line with the un-enhanced conditions, while thresholds in the ITD-based enhancement conditions remained fairly constant.

A two-way repeated measures (RM) analysis of variance (ANOVA) was performed on the three processing conditions (no enhancement, DRR- and ITD-based enhancement) and five distance conditions. Significant main effects of processing [*F*(2,8) = 9.31, *p* = 0.008], and of distance [*F*(4,16) = 29.03, *p* < 0.001] were observed, as well as a significant interaction between enhancement condition and distance [*F*(8,32) = 3.049, *p* = 0.011], indicating that the relative performance for the processing conditions varied across distance. Planned contrasts showed that thresholds in the DRR-based enhancement condition were significantly lower than in the un-enhanced condition [*F*(4,1) = 23.94, *p* = 0.008] as were the thresholds in the ITD-based enhancement condition [*F*(4,1) = 12.78, *p* = 0.023]. A Bonferroni-corrected *post hoc* comparison between the two types of enhancement indicated that the difference in threshold between the DRR- and the ITD-based enhancement conditions (1.089 ± 0.105 *μ*s vs 0.932 ± 0.186 *μ*s, respectively) was not significant (*p* = 0.54).

**Fig. 5:**
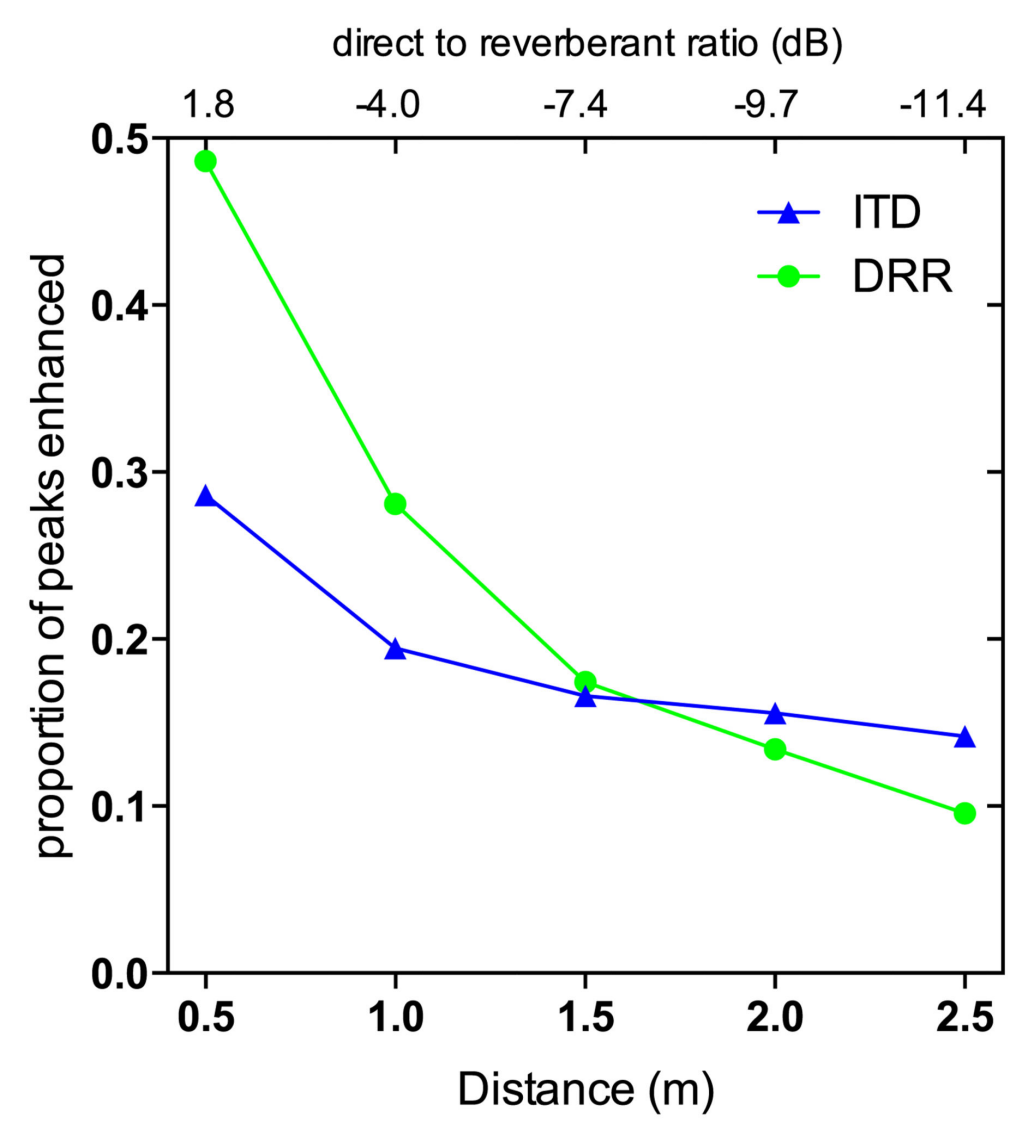
(Color online) Number of peaks selected for enhancement as a function of distance for the DRR- (green circles) and ITD- (blue triangles) based enhancement.

Although the difference in thresholds between the two enhancement methods was not significant overall, there was an apparent decline in performance for the DRR-based enhancement relative to the ITD-based enhancement as distance increased above 1.5 m. Figure 5 shows the proportion of envelope peaks selected for enhancement as a function of distance for both the DRR (green line) and ITD (blue line) based enhancement. Since peak selection in the DRR-based enhancement required moments of positive DRR, and since DRR reduced with distance from the source, fewer peaks were selected for enhancement as distance increased. The decline in the benefit of DRR-based enhancement at greater distances corresponded to the decline in number of peaks enhanced.

In contrast, ITD-based enhancement appeared to provide for a less-marked decline in performance as distance increased and a similar improvement in performance relative to the un-enhanced condition across the range of distances assessed. This may be related to the fact that the number of peaks enhanced was less dependent on DRR in the ITD-based, as compared to the DRR-based, enhancement conditions, and that the ITD-based enhancement explicitly emphasizes moments containing roughly the source ITD.

Overall, ITD discrimination performance was significantly improved by the application of either enhancement method, but this improvement was greatest for source-receiver distances of 1 m and below, which corresponded to DRRs of −4.0 dB and higher. For distances >1 m, the discrimination thresholds were > 1 ms, even after enhancement. Since the maximum direct-sound ITD that can be introduced by microphones worn behind the ears is ~0.8 ms, the practical application of the algorithm may be limited to smaller distances from the source.

## IV Experiment 2: Effect of Enhancement on Extent of Laterality

Experiment 1 demonstrated that enhancement leads to a significant reduction in ITD thresholds. Experiment 2 was designed to assess whether or not the enhancement also generated an overall improvement of horizontal localization ability. Since vocoded stimuli are not heard external to the head, we measured the extent of laterality generated by each ITD. Extent of laterality provides a measure of how far the auditory image of a sound (presented via stereo headphones) deviates from the midline along the interaural axis, somewhat analogous to localization of sources along the left/right dimension in the free field. Both ILDs and ITDs can influence the extent of laterality, and the extent of laterality tends to be smaller for an ITD carried in a stimulus envelope compared to an ITD carried in the TFS (Bernstein and Trahiotis, 1985, 2011b). Increasing envelope modulation depth and “peakiness” have been shown to increase the extent of laterality (Bernstein and Trahiotis, 2003, 2011a,c). The comparison of interest in this experiment was the change in the extent of laterality that was produced for a given ITD for a reverberant sentence with and without enhancement while the direct sound’s ILD was kept zero.

### A Methods

The stimuli were produced in an identical manner to those used in experiment 1. Since the high discrimination thresholds found in the previous experiment indicated that large ITDs would be needed to produce measurable lateralization, ten different ITD conditions were selected that spanned the natural range and beyond: −1.0, −0.8, −0.6 −0.4, −0.2, 0.2, 0.4, 0.6, 0.8, and 1.0 ms, with positive ITDs being right ear leading. For this experiment, participants were asked to indicate the dominant intracranial position of the sound using a visual line pointer, where the line represented the span from the left to the right ear (Seeber and Hafter, 2011). At the start of each trial, the pointer was presented at the midpoint of the line and the participants used a trackball to change its position. The position of the pointer selected by the participant was linearly mapped to a number between −1 and +1. A lateralization of −1 represents an image heard at the left ear and +1 an image heard at the right ear. The order of presentation was randomized across distance and algorithm conditions. As for the ITD discrimination task, interaurally uncorrelated, equally exciting, low-pass noise was presented to prevent low-frequency distortion products from influencing performance. No feedback was given to listeners in this task. Four participants (three female) took part in all conditions, two (including J.J.M.M.) of whom had participated in experiment 1.

### B Results and discussion

**Fig. 6:**
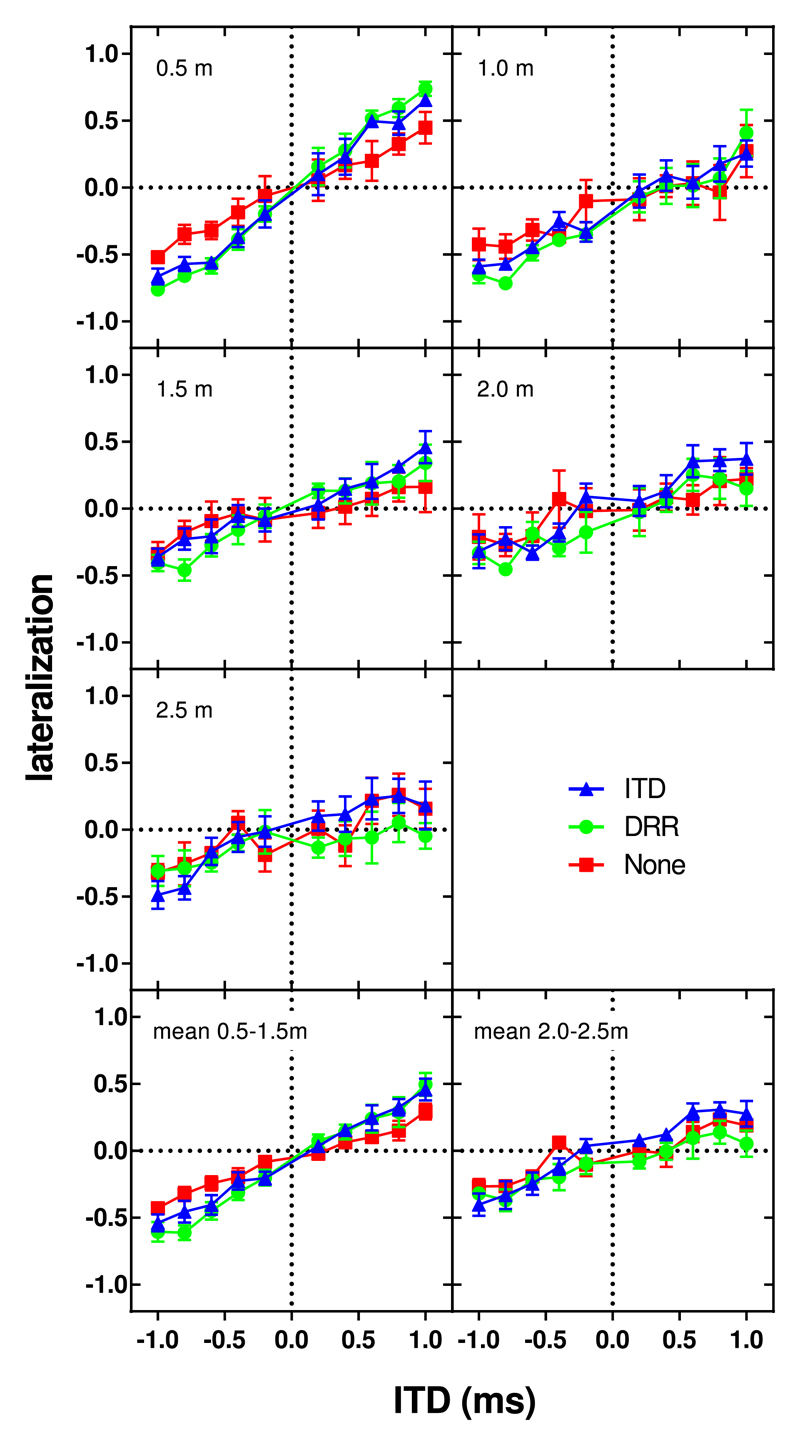
(Color online) Mean extents of laterality for speech stimuli in reverberation as a function of the ITD in the direct sound: DRR- (green circles), ITD- (blue triangles) based enhancement, and un-enhanced (red squares) conditions. Different panels show different distance conditions, and the last two panels show the means across distance conditions 0.5 to 1.5 m and distance conditions 2.0 to 2.5 m. Symbols depict means across participants, error bars show standard errors.

Figure 6 plots the mean extent of laterality across participants as a function of stimulus ITD for each of the enhancement conditions (DRR-based enhancement, the green line with circles; ITD-based enhancement, the blue line with triangles; and no enhancement, the red line with squares). Each distance is plotted in a different panel with the mean across distances separately for 0.5–1 m and 2.0–2.5 m in the two bottom panels.

In general, extent of laterality declined with increasing distance from the source to the receiver. At short distances there was an obvious progression from left to right in perceived location with increasing (negative to positive) ITD, particularly for the enhanced (blue and green symbols), but even for the un-enhanced (red symbols) signals. By 2.0 m, however, lateralization was restricted to ITDs greater than about 0.5 ms. For a distance of 2.5 m, the progression in lateralization was negligible for all but the most extreme ITDs. The extent of laterality was increased at short distances by similar amounts for the ITD and DRR algorithms, compared to the un-enhanced signals.

In the un-enhanced conditions we explored, lateralization was never >±0.52 even for the ITDs of ±1 ms at the closest distance to the source. This maximum lateralization increased to ±0.76 ms for DRR-based enhancement and 0.66 ms for the ITD-based enhancement.

The comparison of interest in this experiment is the change in the gradient of the ITD-lateralization function between the enhanced and unenhanced conditions (Fig. 6). This indicates how the extent of laterality generated for a given ITD differed between conditions. Extent of laterality was modeled with a nested mixed model with fixed effects of ITD, ITD × distance and ITD × distance × (enhancement condition) and random effects of ITD:

(1) $$\text{lat}=c_{0}+c(\text{distance})+\beta (\text{distance})\cdot \text{ITD}+a_{i}+b_{i}\text{ITD},$$

where

$$\beta (\text{distance})=\beta_{1}(\text{distance})+\beta_{2}(\text{distance})\cdot {\text{alg}}_{1}+\beta_{3}(\text{distance})\cdot {\text{alg}}_{2}.$$

In this model, alg1 and alg2 are used to code for the different enhancement methods. alg1 = 1 for the DRR- and ITD-based enhancement conditions and 0 for the no enhancement conditions; alg2 = 1 for the ITD-based enhancement conditions and 0 for DRR-based and no enhancement conditions. As a function of distance, *β*_2_ expresses the general benefit obtained from enhancement while *β*_3_ is sensitive to the difference between the ITD and DRR-algorithms. The random intercept, *a_i_*, and the random effect of ITD, *b_i_*, are different for each participant (indexed by *i*). *c*_0_ is a constant offset from the center-point of the lateralization line, potentially arising from the asymmetric effect of early reflections on the perception of the location of the auditory image. *c* describes how the offset varies as a function of distance from the source.

There were significant main effects of ITD [*χ*^2^(15) = 309.13, *p* < 0.001)] and distance [*χ*^2^(14) = 297.2, *p* < 0.001)]. The interaction between distance and ITD, *β*1, was significant [*F*(4,592) = 8.44, *p* < 0.001], indicating that, as distance from the source increased, extent of laterality for a given ITD decreased. There was a significant change with distance in *c*, the position of the auditory image with no ITD applied [*F*(4,592) = 25.86, *p* < 0.001]. However, over all distances, the intercept *c*_0_ was not significantly different from zero [*F*(1,4) = 0.732, *p* = 0.44].

**Fig. 7:**
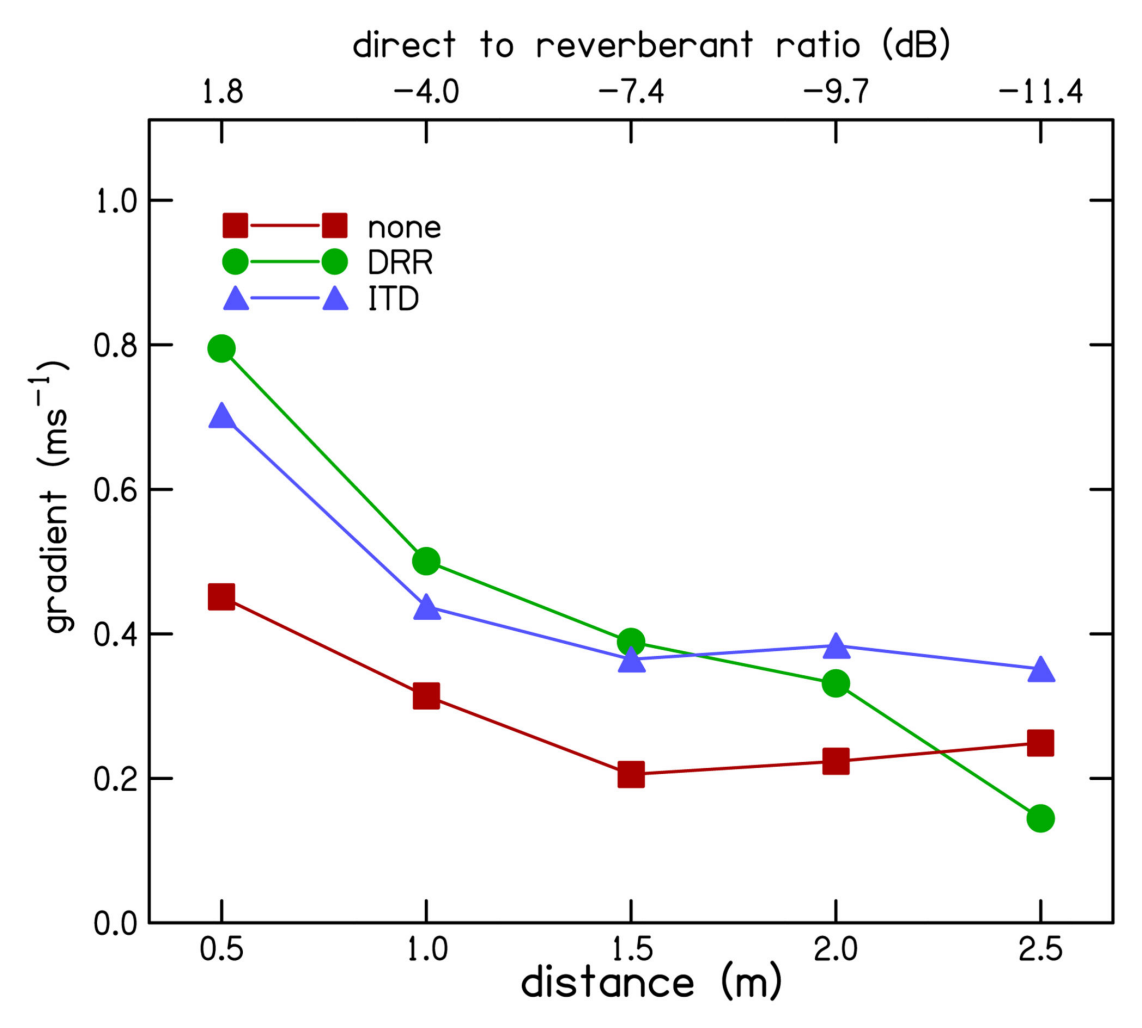
(Color online) Gradient of the ITD-lateralization regression line plotted as a function of virtual source-receiver distance for DRR (green circles) and ITD (blue triangles) based enhancement and the un-enhanced baseline condition (red squares).

We also observed a significant interaction between alg1 and ITD [*F*(1,592) = 43.63, *p* < 0.001] indicating that, over all distances, the extent of laterality produced by a given ITD increased with either form of enhancement compared to the un-enhanced condition. There was also a significant interaction between alg1, ITD, and distance [*F*(4,592) = 11.29, *p* < 0.001], suggesting that the change in the extent of laterality with enhancement for a given ITD differed between distance conditions. The relevant gradient, *β*2, was significantly different from zero, and positive at all distances (apart from 2.5 m). At this distance, *β*2 was significantly negative [*t*(1,592) = −2.154, *p* = 0.032], indicating that the increase in the effect of ITD on extent of laterality with enhancement decreased with increasing distance from the source. There was no significant interaction between alg2 and ITD [*F*(1,592) = 0.549, *p* = 0.459], indicating that there was no overall change in the effect of ITD on extent of laterality for the ITD-based enhancement relative to the DRR-based enhancement. However, there was a significant interaction between alg2, ITD, and distance [*F*(4,592) = 6.08, *p* < 0.001]. For distances of 0.5, 1, and 1.5 m, this gradient, *β*3, was negative, but for 2 and 2.5 m, it was positive. However, *β*3, was only significantly different from zero for a distance of 2.5 m [*t*(1,592) = 4.26, *p* < 0.001] with an estimated value of 0.207. This indicates that, at the furthest tested distance from the source, ITD-based enhancement generated a greater extent of laterality. Figure 7 shows more clearly the change in the gradient of the regression fitted to the ITD-lateralization data with distance for each enhancement condition.

**Fig. 8:**
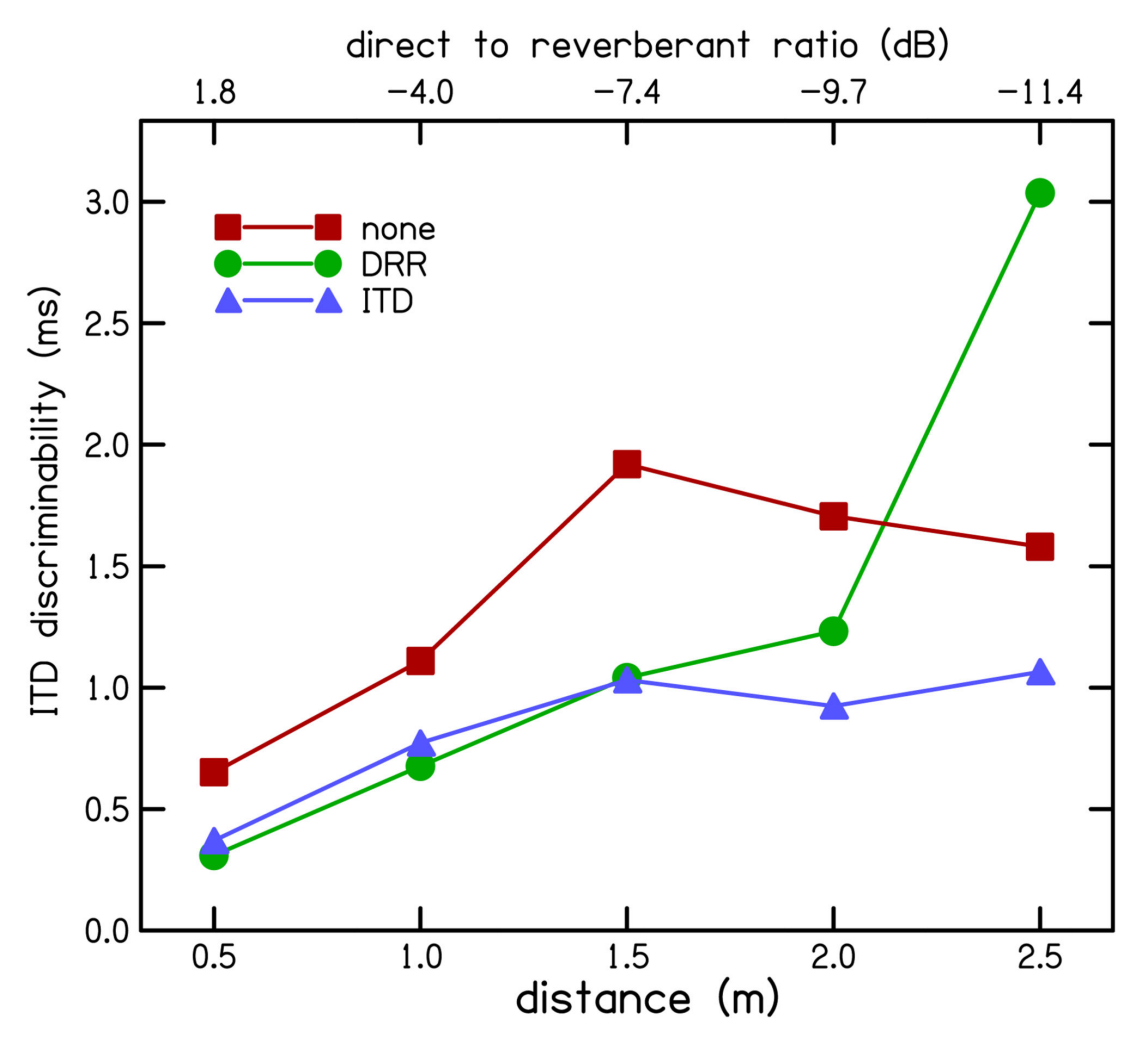
(Color online) ITD discriminability *D*_ITD_ computed from lateralization results for each enhancement condition plotted as a function of distance. ITD discriminability (*D*_ITD_) is defined as the mean standard deviation of the lateralization at each distance divided by the gradient of the lateralization-ITD regression line. *D*_ITD_ is given for DRR- (green circles), ITD- (blue triangles), and un-enhanced (red squares) conditions.

“ITD discriminability” (*D*_ITD_) was defined as in Kerber and Seeber (2013) as the mean standard deviation of the lateralization at each distance divided by the gradient of the lateralization-ITD regression line. This generates a measure similar to the minimum audible angle (MAA; Mills, 1958) but averaged over the range of ITDs tested rather than being defined for a particular reference ITD. It relates to the spatial discrimination in that auditory objects separated by *D*_ITD_ are presumably perceived as occupying different spatial locations. The ITD discriminability *D*ITD for each enhancement condition is plotted as a function of distance in Fig. 8. ITD discriminability for both enhanced conditions was better (smaller) than that for the un-enhanced conditions for all distances except at the greatest distance for which the DRR-based enhancement gave the poorest discriminability. This indicates that enhancement improved the ability to distinguish between two source locations, confirming the findings of experiment 1. The performance of the ITD- and DRR-based enhancement was almost identical except for the largest tested distance where ITD-based enhancement still showed a benefit while performance with DRR-based enhancement declined. The reasons for the latter are not entirely clear. At negative DRRs, the selected peaks might carry an ITD that deviates more from the target ITD, but generally very few peaks are selected for enhancement, effectively converting the DRR-based enhancement algorithm into a no enhancement condition.

## V Experiment 3: Effect of Enhancement on Speech Understanding

Kokkinakis *et al.* (2011) demonstrated that speech intelligibility for CI users decreased exponentially with increasing reverberation time of a room. This is because reverberation smears word boundaries and reduces the amplitude modulation related to the fundamental frequency (F0) of the speech. Poissant *et al.* (2006) showed, using vocoder simulations of bilateral CI listening, that reverberation reduced performance in speech understanding tasks, and that this reduction was significantly greater when only 6, rather than 12, vocoder bands were employed. Ideally, envelope sharpening would improve speech understanding in reverberation by enhancing the saliency of envelope peaks and, by doing so, reinstating temporal cues for speech understanding (although it also has the consequence of introducing additional spectral frequencies to the stimulus). Moreover, since a processing strategy that enhances ITD discrimination at the expense of speech understanding is unlikely to prove beneficial, it was important to determine whether the enhanced processing would degrade speech understanding relative to normal processing. To assess whether speech understanding is maintained following the envelope sharpening employed here, speech understanding was tested using vocoded reverberant speech for each enhancement condition and for each of the five distance conditions assessed in experiments 1 and 2.

### A Methods

The stimuli were generated in the same manner as those for the discrimination and lateralization tasks except that full IEEE sentences were used, and eight, rather than six, vocoder bands were employed in order to increase the amount of low-frequency information provided to aid speech understanding (thus avoiding floor effects on performance). The logarithmic bandwidth of the bands was kept constant and the corner frequency of the lowest band was 1.5 kHz. One sentence list (comprising 10 balanced sentences) from the IEEE sentence corpus (Rothauser *et al.*, 1969) was used for each of the 15 conditions (3 processing conditions × 5 distance conditions), selected pseudorandomly for each participant. None of the sentences had been previously used in experiments 1 and 2. ITDs of ±0.5 ms were applied pseudorandomly to each sentence before vocoding to allow the performance of the algorithms to be assessed under different conditions. Participants listened to the binaurally processed reverberant sentences through headphones and repeated what they heard as accurately as possible. Each sentence had five keywords, and a score was generated for each sentence based on the percentage of keywords correctly identified. The test is open-set so the score for chance performance was negligible. No feedback was given and no low-pass noise was used for this experiment so as not to reduce speech understanding. The sentences were presented at 68 dB SPL (as measured after any enhancement was applied). Five native English speakers (three female) took part in all conditions, three (including J.J.M.M.) of whom took part in experiments 1 and 2.

### B Results and discussion

**Fig. 9:**
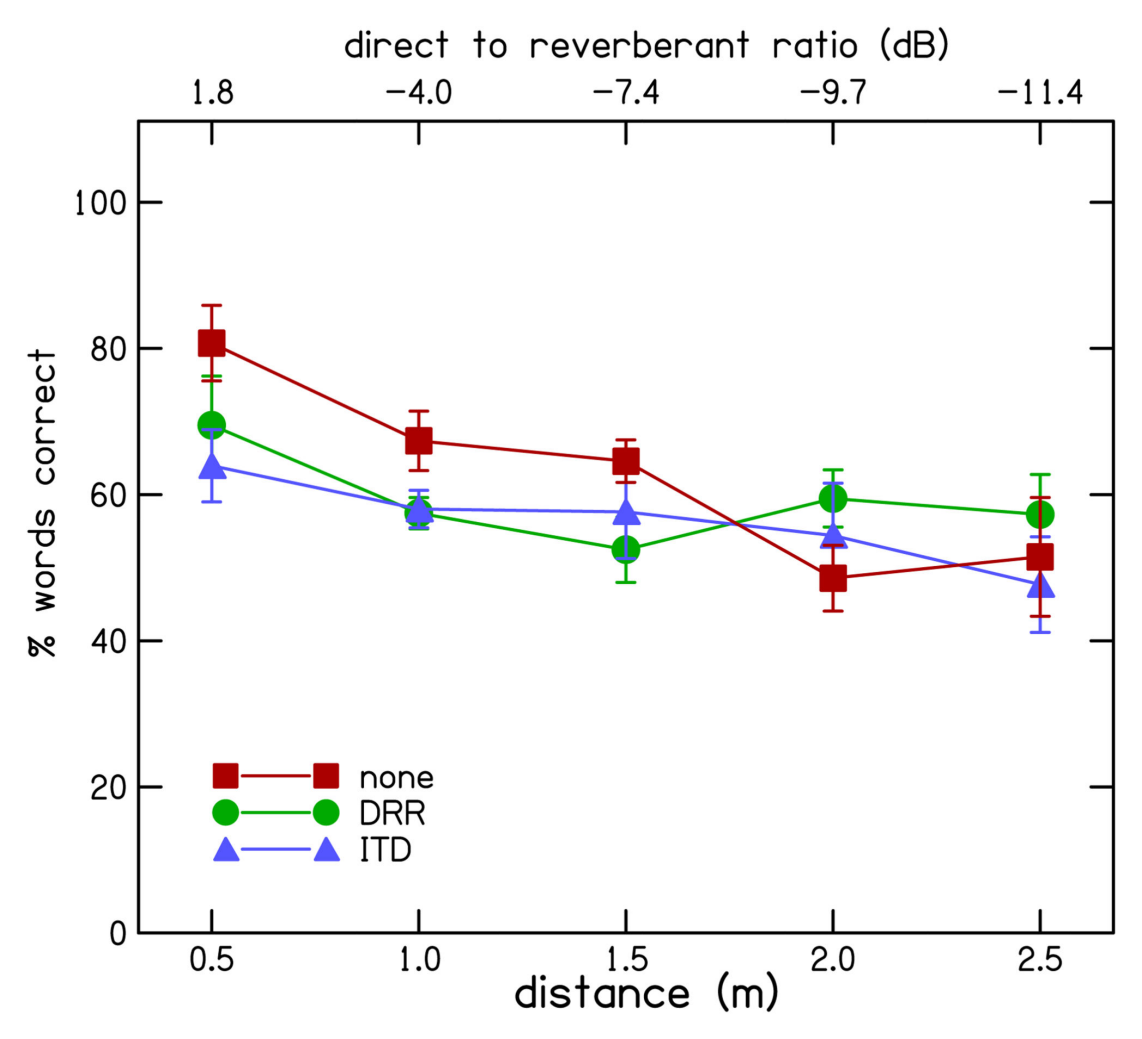
(Color online) Mean percentage across listeners of keywords understood correctly in reverberation as a function of distance from the source. Data are given for DRR-based enhancement (circles and green line), ITD-based enhancement (triangles and blue line), and no enhancement (squares and red line). Error bars show standard errors.

Figure 9 shows the mean percentage of the keywords correctly identified across all participants as a function of distance for each of the enhancement conditions (DRR-based enhancement-green line, ITD-based enhancement-blue line, and no enhancement-red line). As reported previously (Poissant *et al.*, 2006), speech understanding in simulations of CI listening tends to decline with increasing distance from the source, i.e., with increasing relative amounts of reverberation. The mean scores for each enhancement condition averaged over participants and distances were 59.2% for DRR-based enhancement, 56.3% for ITD-based enhancement, and 62.5% for the un-enhanced vocoder.

A two-way repeated measures ANOVA was performed on the three enhancement conditions and five distance conditions. There was no significant main effect of enhancement condition [*F*(2,8) = 2.16, *p* = 0.178]. The 95% confidence intervals for the change relative to the un-enhanced conditions across all distances were −12.6% to 6.0% for the DRR-based enhancement and −14.4% to 2.0% for the ITD-based enhancement. There was a significant main effect of distance [*F*(4,16) = 14.00, *p* < 0.001], indicating that speech understanding declined significantly with distance from the source as DRR reduced. There was also a significant interaction between enhancement condition and distance [*F*(8,32) = 2.64, *p* = 0.024], reflecting the fact that the performance in the enhanced conditions is poorer closer to the source. The reduction in the difference between the enhanced and un-enhanced conditions with distance may have been due to the fact that the number of enhanced peaks decreases with distance (see Fig. 5) so that the stimuli became more like the un-enhanced case, rather than there being a positive increase in the effect of the enhancement with distance. To determine whether there was, in fact, a reduction in intelligibility at shorter distances, planned comparisons between algorithm conditions were performed for the distances 0.5 and 1.0 m, for which the ITD enhancement was most effective. These comparisons revealed that intelligibility was significantly reduced at 1.0 m [*F*(1,4) = 16.52, *p* = 0.031 (Bonferroni corrected for two comparisons)] for the ITD based enhancement, but for the DRR-based enhancement there was no significant difference at either distance.

### C Modified sharpening

Since, for the ITD-based enhancement, speech understanding was significantly reduced when the enhancement algorithms were most active (closest to the source), it is possible that the loss of energy from the peaks after sharpening and bandpass filtering might have been detrimental to speech understanding through reduced DRR. This is because the enhancement truncates half of each selected peak cycle and sets it to zero, thereby reducing the energy of the direct-sound dominated peaks by 3 dB while not affecting the peaks dominated by reverberation.

**Fig. 10:**
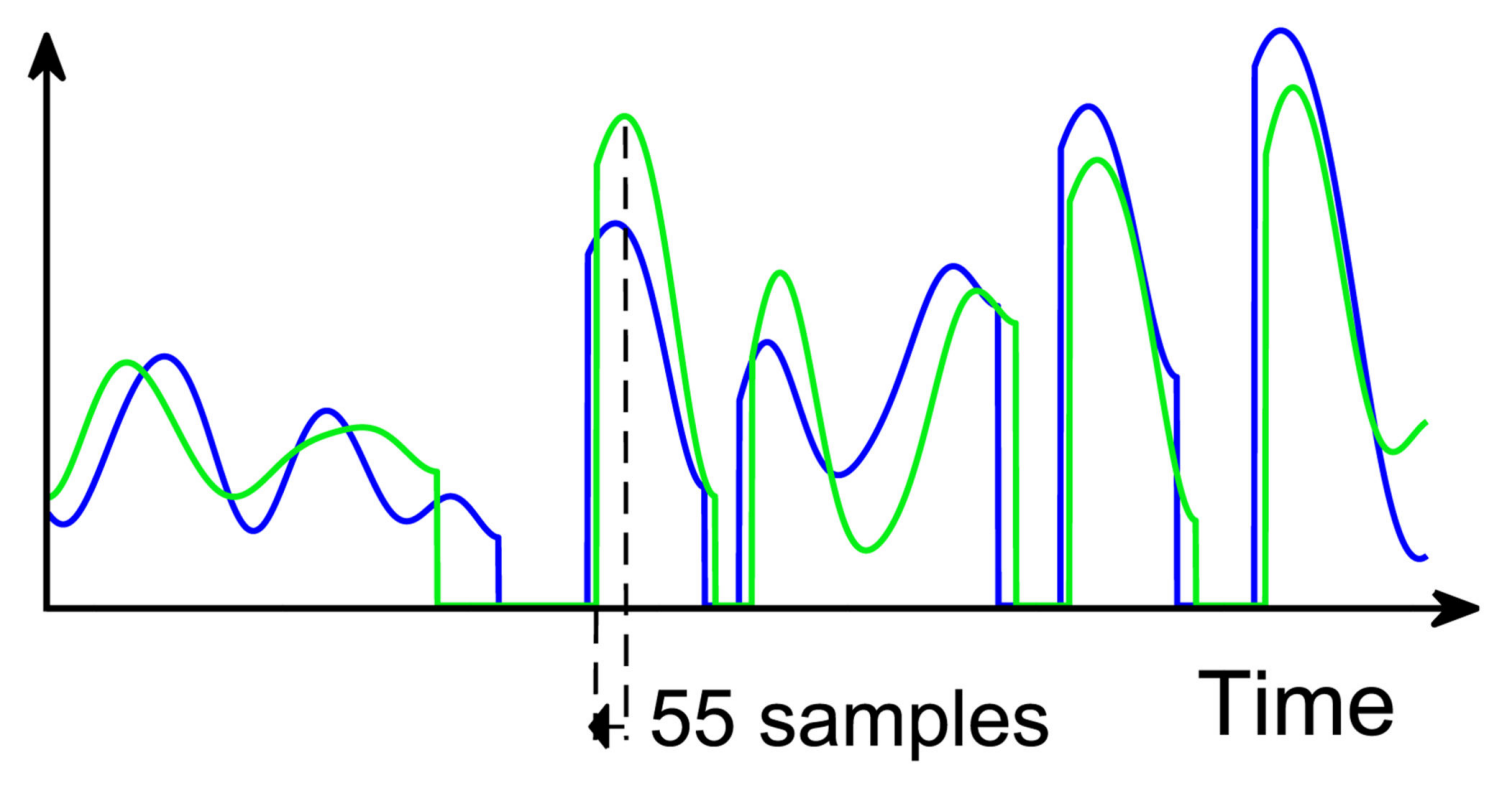
(Color online) Modified sharpening approach. The approach follows that in Fig. 2 except that in (c) the envelope flank is set to zero from 55 samples (1.2 ms) before the peak to the preceding trough rather than directly from the peak. This approach preserves more energy of the selected, direct-sound dominated peaks.

The sharpening method was modified with the aim of reducing the loss of energy from peaks, but without reducing its potential to improve ITD transmission. To this end, rather than setting the whole of the rising flank of selected peaks to zero, the amplitude of the envelope was preserved for a certain number of samples preceding the peak (see Fig. 10). The number of samples that could be preserved without reducing the ITD discrimination performance provided by the enhanced processing was estimated from the performance of S02 (author J.J.M.M.) with an incrementally greater number of samples. The maximum number of samples that could be retained without adversely affecting ITD discrimination performance was estimated to be 55, corresponding to 1.2 ms.

Performance in the speech-understanding task with the modified sharpening was assessed for each enhancement condition and for the five distance conditions. Five native English speakers (three male) who had not taken part in any of the previous three experiments took part in the test.

#### 1 Speech understanding with modified sharpening

**Fig. 11:**
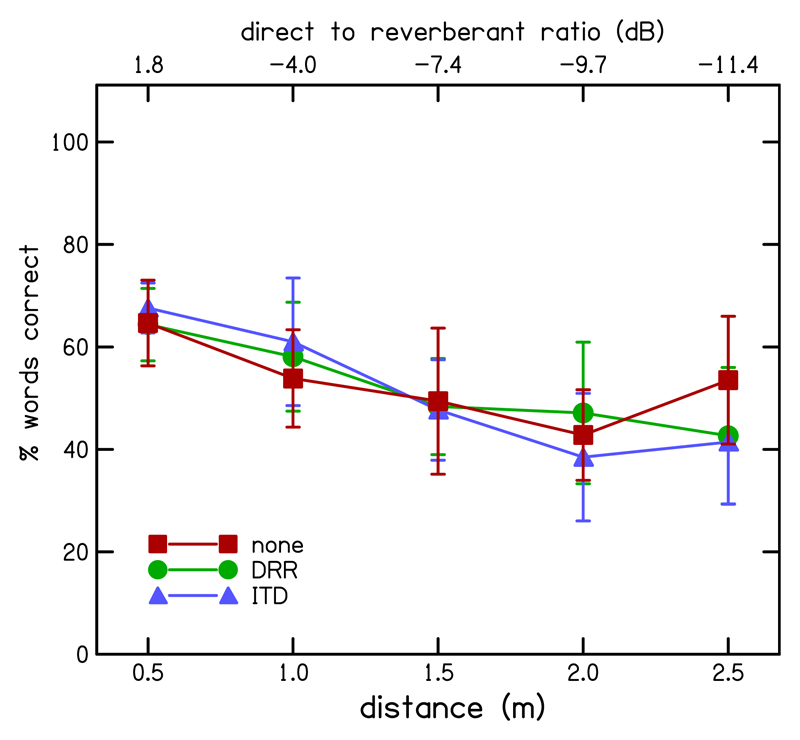
(Color online) Mean percentage of keywords correct across listeners as a function of distance of listener from the source for the modified DRR-based enhancement (circles and green line), modified ITD-based enhancement (triangles and blue line), and no enhancement (squares and red line). Error bars show standard errors.

Figure 11 shows the mean percentage of keywords correctly identified across all participants as a function of distance for each of the enhancement conditions (DRR-based enhancement, green circles; ITD-based enhancement, blue triangles; and no enhancement, red squares). Apart from the slightly higher performance found in the 2.5-m un-enhanced condition, the mean performance for each distance was virtually identical for both enhanced conditions and the unenhanced conditions. Further, performance declined with distance across all enhancement conditions.

A two-way RM ANOVA was performed on the three enhancement conditions and five distance conditions. Mean performance overall for the naive participants was 52.1% for DRR- based enhancement, 51.2% for ITD-based enhancement, and 52.8% for the un-enhanced vocoder. This was 10% lower than that found with the previous, more experienced participants, but again there was no significant difference in performance across the three enhancement conditions [*F*(2,8) = 0.159, *p* = 0.856]. The standard deviation in average performance between participants over all conditions was somewhat larger than that found in the unmodified experiment (21.0% vs 8.3%), due to the large difference in performance between the best two participants and the worst two (74.1% vs 28.9%). The 95% confidence intervals for the change relative to the un-enhanced conditions were −6.5% to 5.0% for the DRR-based enhancement and −11.5% to 8.3% for the ITD-based enhancement. There was still a significant main effect of distance [*F*(4,16) = 11.35, *p* < 0.001], but no significant interaction between enhancement and distance [*F*(8,32) = 1.41, *p* = 0.23]. The only significant difference between the enhancement conditions was between the ITD-based enhancement and un-enhanced conditions at a distance of 2.5 m [*F*(1,4) = 8.99, *p* = 0.04].

In summary, for the modified envelope enhancement neither ITD nor DRR-based enhancement shows a significant detriment.

## VI General Discussion

Based on the findings of Monaghan *et al.* (2013), the feasibility of improving ITD-based lateralization and discrimination in reverberation by selective envelope sharpening was explored. Two methods of identifying optimal moments for sharpening were compared: ITD-based enhancement and DRR-based enhancement, where envelope maxima are selected for sharpening according to the source ITD or a positive DRR. Three experiments were performed to test the effects of selective envelope sharpening on ITD discrimination, lateralization, and speech understanding using a simulation of CI listening. Envelope sharpening significantly improved ITD discrimination and significantly increased the degree of laterality up to twofold at shorter distances from the source. Neither ITD- nor DDR-based enhancement provided significant benefit over the other. Close to the source speech intelligibility was reduced in the ITD-based enhancement conditions. However, speech intelligibility was not reduced or improved by the DRR-based enhancement procedure.

Overall, the results demonstrate that a selective sharpening of envelopes when the direct sound is likely to be dominant produces a significant improvement in spatial hearing in simulations of bilateral CI listening. This demonstrates the success of the method as a proof of concept. Enhancement produced a large improvement in ITD-thresholds close to the source (<1.5 m), but not at greater distances (at least for the DRR-based enhancement). Envelope sharpening also produced significantly greater extents of laterality at all except the largest distance tested (2.5 m). The DRR-based algorithms did not impair speech understanding.

Neither the original enhancement algorithm nor a modified version maintaining more of the direct sound’s energy improved speech intelligibility in reverberation. It is possible that the algorithm would improve speech understanding in competing speech by assisting in spatial segregation. Koning and Wouters (2012) found that a monaural envelope enhancement strategy for CIs that operated by emphasizing the envelope onsets produced increasing speech understanding in noise under some circumstances. Since CI listeners may be relatively unaffected by distortions produced by filter interactions—interactions that can disturb NH participants—it is difficult to determine whether speech understanding in CI listening can be predicted from acoustic simulations. Green *et al.* (2004), for example, found that, whereas a pitch-enhancement algorithm caused NH listeners no difficulties, vowel recognition with CI users was actually reduced. Conversely, noise reduction techniques that reduce intelligibility for NH listeners can actually increase intelligibility for CI users (e.g., Verschuur *et al.*, 2006). It will be necessary to test the envelope enhancement algorithms described in the current study with CI users in order to determine the effect on both ITD perception and speech understanding in reverberation. Some benefit might also accrue employing band selection techniques (Kokkinakis *et al.*, 2011) in conjunction with envelope sharpening to increase speech understanding in reverberation. Potential improvements and additions to the algorithm are discussed below (see Sec. VIC).

Neither enhancement technique (DRR- or ITD-based) proved superior overall to the other in any of the three experiments conducted. At greater source-receiver distances (>1.5 m), ITD-based enhancement had a greater benefit in terms of lower ITD discrimination thresholds and greater extents of laterality, although the thresholds at these distances tended to be too high to prove useful (~1 ms) and extents of laterality remained narrow. ITD-based enhancement reduced speech intelligibility nearer the source, which was not the case for the DRR-based enhancement. With the modified sharpening there was no longer any detriment to speech understanding with either approach. For this modification, the ITD discrimination performance has not been formally tested. Therefore, the technique that might be considered preferable depends only on which is easier to implement. In the current study, the implementation of both enhancement methods made use of prior knowledge. Methods for estimating the relevant parameters are discussed in Secs. VI A and VI B.

### A DRR estimation

A simple way to roughly estimate the DRR is to estimate the reverberant energy as a function of time with a low-pass filter with a time constant aligned to the reverberation time. If the incoming sound’s energy exceeds that of the estimated reverberant energy, the presumed DRR is positive and the peak is selected for enhancement. The reverberation time can be estimated from the signal’s decay time after a peak. The advantage of this approach is that it works independently on each ear.

When binaural signals are available, a measure expected to correlate with signal DRR is binaural coherence. The direct sound is almost fully coherent; as reflections build up and interfere, the signal coherence reduces. This underlies the strategy of Faller and Merimaa (2004) in estimating the ITD and ILD cues used by listeners in noisy or reverberant environments. The assumption is that humans exploit ITD and ILD cues only in highly coherent parts of the signal. Coherence has also been used in recent techniques for estimating the BRIR (e.g., Jeub *et al.*, 2011; Tsilfidis *et al.*, 2011; Tsilfidis and Mourjopoulos, 2012), and Cosentino *et al.* (2012) used coherence to estimate when the DRR of a reverberant signal was high. Similarly, kurtosis has been shown to decrease with increasing reverberation (Wu and Wang, 2006), and a kurtosis-like feature was employed by Hazrati *et al.* (2013) to generate an estimated binary mask algorithm that improved monaural understanding of speech in reverberation by CI listeners. An advantage of a DRR-related decision criterion is that it extends easily to situations with multiple sources since it is independent of the number and spatial distribution of sound sources.

### B ITD estimation

Although the average ITD of a signal can be calculated from the location of the maximum of the cross correlation of the left and right channels of the signal, in order to separate the source from reflections, it is necessary to estimate the ITD of the sound source alone, i.e., the direct-sound ITD. This is more difficult as the direct sound is often lower in level than the reverberant sound, which, as a result, dominates the cross correlation. A simple calculation of the ITD will tend to suggest an ITD of zero when the reverberation dominates, as the reverberant sound field tends to be isotropic. As above, coherence (or kurtosis) can be used to estimate which parts of the signal give rise to the most direct sound energy. By weighting the cross correlation by the coherence of the windowed signal, an accurate estimate of the source ITD can be obtained (Faller and Merimaa, 2004). A reasonable strategy would be to update the target ITD only when a reliable estimate is available. The approach could be extended to multiple sources by selecting peaks belonging to more than one ITD. Boyd *et al.* (2013) demonstrated that the GCC-PHAT (generalized cross correlation with phase transform) algorithm can be used to obtain accurate ITDs from multiple sources in the presence of reverberation.

### C Combining the enhancement approach with other techniques

The sharpening algorithm does not appear to increase speech understanding, and perhaps the greatest benefits will be realized by combining the sharpening approach to improve spatial localization with other algorithms that have been found to increase intelligibility of speech in reverberation. Kokkinakis *et al.* (2011) used a frequency band selection method to improve speech understanding in bilateral CI patients in reverberation. The method involved selecting time-frequency frames with DRR >-5 dB, assuming prior knowledge of the direct and reverberant energy. Cosentino *et al.* (2012) modified this method by using binaural coherence as a correlate of the DRR of the reverberant speech and identifying an optimum coherence threshold for each frequency band. A binary mask was applied to frequency-time bins so that all bins that did not meet the threshold criterion were set to zero. This was found to enhance reverberant speech understanding in CI simulations. Similarly, Hazrati *et al.* (2013) used a monaural, kurtosis-like feature to estimate the binary mask and found an improvement in monaural speech understanding for CI listeners in reverberation. It seems plausible that sharpening the peaks in the time-frequency bins with low levels of reverberation as identified by the algorithms of Cosentino *et al.* (2012) or Hazrati *et al.* (2013) would lead to improved localization ability, as well as increased speech understanding.

### D Applicability of results to bilateral cochlear implantation

Assessing NH listeners using vocoded signals represents an idealized and restricted mean by which the information available to CI users may be studied. A remaining question concerns the extent to which improvements provided by the enhancement approach studied with vocoder simulation of CI listening with NH listeners might transfer to a benefit for CI users. A natural future direction for this project, therefore, would be to test the algorithm with CI users, both to determine whether it can improve the use of ITD cues under reverberant conditions and to determine whether or not it affects speech understanding.

Although the stimulus conveyed in acoustic simulations is similar to that presented to electrodes in electrical hearing, the neural response evoked can differ significantly. It is not necessarily the case, therefore, that envelope manipulations that prove beneficial to ITD sensitivity in acoustic hearing will have the same effect in electric hearing. However, the few physiological and psychophysical studies on the effect of envelope parameters on ITD sensitivity with electrical stimulation demonstrate similar trends to those employing acoustic stimuli. In a study of NH and deafened, bilaterally implanted ferrets, Hartley and Isaiah (2014) compared neural ITD sensitivity for sinusoidal amplitude modulated (SAM) envelopes and half-wave rectified (HWR) envelopes. They found that ITD sensitivity was greater for HWR envelopes, which are both steeper than SAM and have a greater off-time. The only study of envelope parameters to date to employ both CI and NH listeners (with acoustic stimulations) is that of Laback *et al.* (2011). The study used trapezoid envelope shapes to investigate the effect of off-time, onset gradient and peak level on envelope ITD discrimination thresholds. These authors found that, in contrast to NH subjects, CI listeners showed, on average, no effect of the onset gradient for the range tested (6% dynamic range/ms to 24% dynamic range/ms). If the gradient of the envelope is indeed less important for CI listeners, this presents a challenge to the prospect of transferring the benefit of the sharpening algorithm found in NH listeners to CI users. However, slopes may need to be considerably steeper than those tested, which is supported by the fact that the CI listeners tested with steeper gradients of 28% dynamic range/ms generally showed best performance in that condition. Also, in a separate pulsatile condition (albeit with a higher current level), Laback *et al.* (2011) found much lower thresholds when slopes were effectively infinite. Additionally it was observed that, in both NH listeners and CI users, longer off-times resulted in lower ITD thresholds. Since one of the effects of our sharpening technique was to increase the envelope off-time, CI users may benefit from the algorithm even if increasing the gradient itself is not beneficial. However, as seen in experiment 3, increasing envelope off-time may also reduce speech intelligibility.

## VII Summary and Conclusions

The findings of Monaghan *et al.* (2013) motivated the desire to devise a method by which the representation of envelope ITD cues in reverberation might be enhanced through the selective sharpening of stimulus envelopes, i.e., increasing modulation depth and onset gradient. The current study, therefore, described two methods of identifying those regions of the stimulus that might be appropriate for such sharpening (i.e., appropriate with respect to the amount of information concerning the direct sound present in that region of the stimulus waveform). Since enhancement of envelope ITD is likely to be most advantageous to CI users, the algorithms were tested using a simulation of listening in a reverberant environment with bilateral CIs. Three experiments were undertaken to determine the effect of the sharpening algorithms on vocoded listening. These experiments demonstrated that selectively sharpening stimulus envelopes can improve ITD discrimination and the extent of laterality of a sound. In the case of the DRR-based enhancement, there was no apparent reduction in speech understanding. For the ITD-based enhancement technique speech intelligibility was reduced close to the source, where enhancement was most active. A modified version of the sharpening technique that retained more energy at the enhanced peaks was tested and found to have no effect on speech in either the ITD- or DRR-based enhancement conditions. However, ITD discrimination was not formally assessed for the modified sharpening. Overall, neither method of selection was found to be superior to the other. Applying strategies in hearing devices to enhance the envelope, thus, ensuring the minimal conditions for ITD discrimination in stimulus envelopes, could lead to improvements in localization performance in reverberant spaces. The concept of enhancing the saliency of ITD cues in reverberation, rather than merely trying to reduce the amplitude of the reverberant energy, is novel, and the data presented here indicate the promise of this approach. The benefit for lateralization is substantial in that the lateralization slope increased about twofold, but the benefit was limited mainly to a DRR of better than −3 dB. The combination of this enhancement technique with other methods of identifying the direct sound components of the signal to reduce the effect of reverberant energy may greatly increase the benefits of both techniques.

In its current instantiation, the algorithm requires *a priori* information regarding the room acoustics, but future developments should allow it to predict the necessary information with a high degree of accuracy. Based on these tests of the envelope enhancement approach with vocoders, it can be hoped that ITD sensitivity and lateralization can be improved with bilateral CIs using the presented approach.
